# Complete Genome Sequences of *Helicobacter pylori* Clarithromycin-Resistant Strains

**DOI:** 10.1128/genomeA.00912-13

**Published:** 2013-11-14

**Authors:** Tran Thanh Binh, Rumiko Suzuki, Seiji Shiota, Dong Hyeon Kwon, Yoshio Yamaoka

**Affiliations:** Department of Environmental and Preventive Medicine, Oita University Faculty of Medicine, Yufu, Japana; Biology Department, Long Island University, Brooklyn, New York, USAb; Department of Medicine-Gastroenterology, Baylor College of Medicine and Michael E. DeBakey Veterans Affairs Medical Center, Houston, Texas, USAc

## Abstract

We report the complete genome sequences of two *Helicobacter pylori* clarithromycin-resistant strains. Clarithromycin (CLR)-resistant strains were obtained under the exposure of *H. pylori* strain 26695 on agar plates with low clarithromycin concentrations. The genome data provide insights into the genomic changes of *H. pylori* under selection by clarithromycin *in vitro*.

## GENOME ANNOUNCEMENT

*Helicobacter pylori* is a spiral Gram-negative bacterium that infects more than half of the world’s population and is currently accepted to play a causative role in the pathogenesis of chronic gastritis, peptic ulcer diseases, gastric cancer, and mucosa-associated lymphoid tissue (MALT) lymphoma (1, 2). The eradication of *H. pylori* not only improves peptic ulcer healing but also prevents its recurrence and reduces the risk of developing gastric cancer (3–6). However, the prevalence of antibiotic resistance in *H. pylori* is increasing worldwide and is a growing public health problem needing more attention (7–11). Especially, resistance to clarithromycin (CLR) is the most important factor causing treatment failure and is responsible for the decline of the *H. pylori* eradication rate (7, 12–14).

In the present report, we announce two genome sequences of CLR-resistant *H. pylori* strains (26695-1CL and 26695-1CH) obtained *in vitro*. The genomes of these strains provide insights into the genomic changes of *H. pylori* under CLR selection.

Two CLR-resistant strains were obtained under exposure to low concentrations of CLR *in vitro* (0.03 and 0.06 µg/ml CLR agar plates), and whole-genome sequencing was performed using 90-base paired-end reads on the Illumina HiSeq 2000 genome sequencer (Illumina, Inc., San Diego, CA). The whole-genome sequences of the two strains were reconstructed by mapping the short-read sequences on the genome of *H. pylori* 26695 (GenBank accession no. NC_000915) using the CLC Genomics Workbench version 4.0 (CLC bio, Aarhus, Denmark). The lengths of the 26695-1CL and 26695-1CH genomes are 1,667,239 bp and 1,667,302 bp, with coverage depths of 840× and 1,257×, respectively. The G+C content of both strains is 38.87%.

In the two CLR-resistant strains, 5 and 7 mutations (including the well-known mutations in the 23S rRNA gene) were found. Three mutations (C712T, T743A, and G160A in the genes *hp0190*, *hp0471*, and *hp1048*, respectively) were present in both strains. Two mutations were found only in the *H. pylori* 26695-1CL strain, without mutations in the 23S rRNA gene, and four mutations were found only in the *H. pylori* 26695-1CH strain, including the A2143G mutation in the 23S rRNA gene.

### Nucleotide sequence accession numbers.

The genome sequences of clarithromycin-resistant *H. pylori* strains 26695-1CL and 26695-1CH were deposited at GenBank under the accession no. AP013356 and AP013355, respectively.

## References

[1] Peek RM Jr Blaser MJ 2002 *Helicobacter pylori* and gastrointestinal tract adenocarcinomas. Nat. Rev. Cancer 2:28–37 11902583 10.1038/nrc703

[2] Suerbaum S Michetti P 2002 *Helicobacter pylori* infection. N. Engl. J. Med. 347:1175–1186 12374879 10.1056/NEJMra020542

[3] Hosking SW Ling TK Chung SC Yung MY Cheng AF Sung JJ Li AK 1994 Duodenal ulcer healing by eradication of *Helicobacter pylori* without anti-acid treatment: randomised controlled trial. Lancet 343:508–510 7906759 10.1016/s0140-6736(94)91460-5

[4] Shiota S Yamaoka Y 2010 Management of *Helicobacter pylori*. F1000 Med. Rep. 2:20–22 10.3410/M2-20 PMC2948387 20948865

[5] Takenaka R Okada H Kato J Makidono C Hori S Kawahara Y Miyoshi M Yumoto E Imagawa A Toyokawa T Sakaguchi K Shiratori Y 2007 *Helicobacter pylori* eradication reduced the incidence of gastric cancer, especially of the intestinal type. Aliment. Pharmacol. Ther. 25:805–812 17373919 10.1111/j.1365-2036.2007.03268.x

[6] Malfertheiner P Megraud F O’Morain C Bazzoli F El-Omar E Graham D Hunt R Rokkas T Vakil N Kuipers EJ 2007 Current concepts in the management of *Helicobacter pylori* infection: the Maastricht III Consensus Report. Gut 56:772–781 17170018 10.1136/gut.2006.101634 PMC1954853

[7] Mégraud F 2004 *H pylori* antibiotic resistance: prevalence, importance, and advances in testing. Gut 53:1374–1384 15306603 10.1136/gut.2003.022111 PMC1774187

[8] Debets-Ossenkopp YJ Herscheid AJ Pot RG Kuipers EJ Kusters JG Vandenbroucke-Grauls CM 1999 Prevalence of *Helicobacter pylori* resistance to metronidazole, clarithromycin, amoxicillin, tetracycline and trovafloxacin in The Netherlands. J. Antimicrob. Chemother. 43:511–515 10350380 10.1093/jac/43.4.511

[9] Fock KM Katelaris P Sugano K Ang TL Hunt R Talley NJ Lam SK Xiao SD Tan HJ Wu CY Jung HC Hoang BH Kachintorn U Goh KL Chiba T Rani AA Second Asia-Pacific Conference 2009 Second Asia-Pacific consensus guidelines for *Helicobacter pylori* infection. J. Gastroenterol. Hepatol. 24:1587–1600 10.1111/j.1440-1746.2009.05982.x 19788600

[10] Glupczynski Y Mégraud F Lopez-Brea M Andersen LP 2001 European multicentre survey of in vitro antimicrobial resistance in *Helicobacter pylori*. Eur. J. Clin. Microbiol. Infect. Dis. 20:820–823 11783701 10.1007/s100960100611

[11] Malfertheiner P Megraud F O’Morain CA Atherton J Axon AT Bazzoli F Gensini GF Gisbert JP Graham DY Rokkas T El-Omar EM Kuipers EJ European Helicobacter Study Group 2012 Management of *Helicobacter pylori* infection—the Maastricht IV/ Florence Consensus Report. Gut 61:646–664 22491499 10.1136/gutjnl-2012-302084

[12] Jenks PJ 2002 Causes of failure of eradication of *Helicobacter pylori*. BMJ 325:3–4 12098709 10.1136/bmj.325.7354.3 PMC1123549

[13] Mégraud F Lamouliatte H 2003 Review article: the treatment of refractory *Helicobacter pylori* infection. Aliment. Pharmacol. Ther. 17:1333–1343 12786627 10.1046/j.1365-2036.2003.01592.x

[14] Vakil N Megraud F 2007 Eradication therapy for *Helicobacter pylori*. Gastroenterology 133:985–1001 17854602 10.1053/j.gastro.2007.07.008

